# Creating assessments as an active learning strategy: too time-consuming for benefit gained?

**DOI:** 10.1080/10872981.2019.1649572

**Published:** 2019-07-26

**Authors:** Emily Kalms

**Affiliations:** Imperial College London

Dear Editor,

The study by Kurtz et al. [1] shed light on how students can benefit from writing multiple choice questions (MCQs) as an active learning strategy. As students increasingly learn by answering practice questions online [2], devising and sharing questions could provide further benefit. As an Associate Fellow of the Higher Education Academy, I have seen the value of students answering questions to revise but haven’t seen peer-devised question banks shared amongst my fellow medical students.

The mixed methods study highlighted that students found writing questions a beneficial tool [1]. However, there were concerns that this was time-consuming and, for this reason, students should have maximum benefit from questions they design. This could be achieved by involving a large cohort to devise questions to share in a peer-made question bank. Moreover, existing online question banks aren’t targeted to specific curricula [2] so students may benefit from resources focussing on their institution’s learning objectives.

Maximum benefit from these devised questions would require questions to cover universally challenging themes identified by students. As question authors have researched their difficult topic in detail, relevant information found should be included alongside answers. Students benefit from collaborative reviews [1] so may appreciate their peers’ explanations of problematic areas. As Kurtz et al. [1] mentioned, faculty members should review questions and explanations to ensure errors aren’t shared.

In addition to concerns about time spent writing questions, there may be limitations with different question styles. Imperial College School of Medicine regularly tests students with Single Best Answer questions (SBAs) rather than MCQs. SBAs are an excellent tool to consider similar differentials and focus on the most likely and ‘best’ option. However, developing these questions is difficult and students may have insufficient knowledge to consider which option is first-line and appropriate plausible detractors [3]. However, the question types have similarities and include pathognomonic ‘clues’ so developing MCQs may still benefit students sitting SBA questions.

To conclude, as highlighted by Kurtz et al [1], creating assessments, particularly with MCQs, can be beneficial for students’ active learning. Students find revising topics through practising questions useful [2] and therefore could benefit further from designing questions. However, further research is needed to ascertain if this would be beneficial for other question styles e.g. SBAs. Furthermore, due to exercise’s time-consuming nature, benefit to students must be optimised with questions focussing on difficult topics, targeted to specific curricula and shared amongst students.
